# Hospitalization costs of severe bacterial pneumonia in children: comparative analysis considering different costing methods

**DOI:** 10.1590/S1679-45082017GS3855

**Published:** 2017

**Authors:** Sheila Elke Araujo Nunes, Ruth Minamisava, Maria Aparecida da Silva Vieira, Alexander Itria, Vicente Porfirio Pessoa, Ana Lúcia Sampaio Sgambatti de Andrade, Cristiana Maria Toscano

**Affiliations:** 1 Universidade Estadual da Região Tocantina do Maranhão, Imperatriz, MA, Brazil.; 2 Universidade Federal de Goiás, Goiânia, GO, Brazil.; 3 Pontifícia Universidade Católica de Goiás, Goiânia, GO, Brazil.; 4 Hospital da Criança, Goiânia, GO, Brazil.

**Keywords:** Pneumonia, Hospitalization, Child, Costs and cost analysis, Health expenditures, Medical records, Unified Health System

## Abstract

**Objective:**

To determine and compare hospitalization costs of bacterial community-acquired pneumonia cases via different costing methods under the Brazilian Public Unified Health System perspective.

**Methods:**

Cost-of-illness study based on primary data collected from a sample of 59 children aged between 28 days and 35 months and hospitalized due to bacterial pneumonia. Direct medical and non-medical costs were considered and three costing methods employed: micro-costing based on medical record review, micro-costing based on therapeutic guidelines and gross-costing based on the Brazilian Public Unified Health System reimbursement rates. Costs estimates obtained via different methods were compared using the Friedman test.

**Results:**

Cost estimates of inpatient cases of severe pneumonia amounted to R$ 780,70/$Int. 858.7 (medical record review), R$ 641,90/$Int. 706.90 (therapeutic guidelines) and R$ 594,80/$Int. 654.28 (Brazilian Public Unified Health System reimbursement rates). Costs estimated via micro-costing (medical record review or therapeutic guidelines) did not differ significantly (p=0.405), while estimates based on reimbursement rates were significantly lower compared to estimates based on therapeutic guidelines (p<0.001) or record review (p=0.006).

**Conclusion:**

Brazilian Public Unified Health System costs estimated via different costing methods differ significantly, with gross-costing yielding lower cost estimates. Given costs estimated by different micro-costing methods are similar and costing methods based on therapeutic guidelines are easier to apply and less expensive, this method may be a valuable alternative for estimation of hospitalization costs of bacterial community-acquired pneumonia in children.

## RESUMO

**Objetivo:**

Determinar e comparar custos hospitalares no tratamento da pneumonia bacteriana adquirida na comunidade por diferentes metodologias de custeio, na perspectiva do Sistema Único de Saúde.

**Métodos:**

Estudo de custo, com coleta de dados primários de uma amostra de 59 crianças com 28 dias a 35 meses de idade hospitalizadas por pneumonia bacteriana. Foram considerados custos diretos médicos e não médicos. Três metodologias de custeio foram utilizadas: microcusteio por revisão de prontuários, microcusteio considerando diretriz terapêutica e macrocusteio por ressarcimento do Sistema Único de Saúde. Os custos estimados pelas diferentes metodologias foram comparados utilizando o teste de Friedman.

**Resultados:**

Os custos hospitalares de crianças com pneumonia grave foram R$ 780,70 ($Int. 858.7) por revisão de prontuários, R$ 641,90 ($Int. 706.90) por diretriz terapêutica e R$ 594,80 ($Int. 654.28) por ressarcimento do Sistema Único de Saúde, respectivamente. A utilização de metodologias de microcusteio (revisão de prontuários e diretriz) resultou em estimativas de custos equivalentes (p=0,405), enquanto o custo estimado por ressarcimento foi significativamente menor do que aqueles estimados por diretriz (p<0,001) e por revisão de prontuário (p=0,006), sendo, assim, significativamente diferentes.

**Conclusão:**

Na perspectiva do Sistema Único de Saúde, existe diferença significativa nos custos estimados quando se utilizam diferentes metodologias, sendo a estimativa por ressarcimento a que resulta em valores menores. Considerando que não há diferença nos valores de custos estimados por diferentes metodologias de microcusteio, a metodologia de custeio por diretriz, de mais fácil e rápida execução, é uma alternativa válida para estimativa de custos de hospitalização por pneumonias bacterianas em crianças.

## INTRODUCTION

Pneumonia is a significant cause of morbidity and mortality all over the world. In 2010, an estimated 120 million new episodes of the disease affected children aged under 5 years, with 935 thousand cases progressing to death worldwide.^1^ In Latin America, 980 thousand to 1.5 million cases of pneumonia are estimated to occur in children aged under 5 years each year.^2-4^ Brazil is one of the countries with high incidence of pneumonia worldwide.^5^

Estimated costs of inpatient treatment of pneumonia in children aged less than 5 years in Latin America range from US$ 804.46 to US$ 1,076.89.^2,6^ Several costing methods with different levels of accuracy and quality may be applied to estimate disease costs, bottom-up micro-costing, top-down micro-costing and gross-costing being the most common*.* ^7^

Pneumonia represents a high economic burden to society and the health care system.^2,8^ The Brazilian Public Unified Health System (SUS - *Sistema Único de Saúde*) covers about 75% of the population; therefore, estimating pediatric pneumonia costs under the SUS perspective is important. Given the variability of results across costing methods, estimation of Brazilian costs of inpatient cases of severe pneumonia in children via different methods is of particular relevance.

## OBJECTIVE

To determine and compare *Sistema Único de Saúde* hospital costs to treat bacterial community-acquired pneumonia cases estimated via different costing methods.

## METHODS

### Study design, location and period

An observational descriptive partial economic evaluation study under the SUS perspective. Costs of the disease were estimated using different methods. Costing studies were carried out in Goiânia (GO) between October and December 2011.

### Study population and sample

The study population comprised children aged between 28 days and 35 months hospitalized due to community-acquired pneumonia (CAP), and enrolled in a prospective population-based pneumonia surveillance study conducted in 17 child hospitals in the city of Goiânia (GO).^8^ This costing study was based on a sample of children admitted to two of these hospitals, totaling up 111 clinical beds and 38 pediatric intensive care unit (ICU) beds, accounting to approximately 30% of children hospitalized due to CAP, in a prospective population-based design. Selected cases corresponded to children hospitalized due to suspected pneumonia (cough and/or dyspnea) with clinical or radiological confirmation and receiving antimicrobial therapy during the period of admission. Cases with a discharge diagnosis of viral pneumonia, bronchiolitis, asthma or atypical pneumonia, as well as those involving patients admitted through health insurance plans were excluded.

Over the course of the study period, 1,520 children with suspected pneumonia were identified via the surveillance study, 330 of which met CAP definition criteria. Of these, 106 (32%) were admitted to the two hospitals included in this study, of which 11 (10%) were excluded due to missing records. Out of 95 children whose medical records were evaluated, 11 (12%) were excluded due to lack of antimicrobial therapy over the course of hospital stay, 5 (5%) due to diagnosis upon discharge of viral pneumonia, bronchiolitis, asthma or atypical pneumonia, and 31 (31%) due to admission costs paid by a health insurance plan. The final sample comprised 59 cases of children with pneumonia and admitted through the SUS, in that, 52 children considered severe cases and treated in inpatient units, and 7 graded as very severe and treated at ICU.

### Cost components

In compliance with international recommendations,^7,9,10^ direct costs were considered, including medical and non-medical costs used in previous published studies on pneumonia costs.^2,10-12^ Medical costs included hospital services (admission to ward and/or ICU), professional services (medical fees), drugs, radiological examinations and laboratory tests, and respiratory physical therapy. Non-medical costs comprised daily companion charge rates. Data concerning healthcare resource utilization were analyzed from date of admission to date of discharge.

Costs were calculated in *reals* (R$; December 2011) and converted to international dollars ($Int.) at the rate of R$ (*Real*) 1,00=$Int. 1.10; 2011 exchange rates).^13^

### Costing methods

Three costing methods were used in this study: (1) bottom-up micro-costing (cost estimation based on medical record review); (2) top-down micro-costing, (cost estimation based on therapeutic guidelines); and (3) top-down gross-costing (cost estimation based on SUS reimbursements rates to hospital service providers).

For costing purposes, inpatient ward and ICU length of stay were taken into account in cases of very severe pneumonia treated at both levels. Procedures not listed in SUS reimbursement tables and which therefore did not have a predefined reimbursement rate (*e.g*., suction, nebulization and oxygen supply) were not taken into account.

### Costing based on medical record review

Data concerning healthcare resource utilization were extracted from medical records. Medical costs included hospital services, medical fees, drugs and procedures; non-medical costs corresponded to daily companion charge rates (SUS charge rate, R$ 8,00).

Unitary prices of individual resources consumed were multiplied by the estimated amount of resources. Drug costs per child were calculated according to prescribed doses administered over the curse of hospital stay. Drug prices were based on average prices listed in *Banco de Preços em Saúde* (BPS),^14^ *Câmara de Regulação do Mercado de Medicamentos* (CMED)^15^ and *Revista ABC Farma*.^16^ Prices of similar drugs manufactured by different pharmaceutical industries vary; therefore, drug prices in this study were based on the formulary of one of the hospitals considered, and the 17% Goiás State tax (*Imposto sobre Circulação de Mercadorias e Serviços -* ICMS) was calculated.

Costs of professional and hospital services, respiratory physical therapy, radiological examinations and laboratory tests were estimated based on *Sistema de Gerenciamento da Tabela de Procedimentos, Medicamentos, Órteses, Próteses e Materiais Especiais do* SUS (SIGTAP),^17^ or “SUS table”.

Daily hospital bed rates and medical fees were estimated by dividing respective SUS reimbursement rates by patient average length of stay.

### Costing based on therapeutic guidelines

Costs of health resources consumed per patient were estimated according to standard therapeutic guidelines given by the *Sociedade Brasileira de Pneumologia e Tisiologia* and *Sociedade Brasileira de Pediatria* for treatment of children hospitalized due to CAP^18^ (Chart 1).

**Chart 1:** Health resources considered for therapeutic-guideline-based costing of community-acquired pneumonia

Components	Health resources
Drugs*	Children <2 months: ampicillin (50mg/kg/day) + gentamicin (7,5mg/kg/day) and injectable dipyrone Children ≥2 months: crystalline penicillin (50,000IU/kg/day) and injectable dipyrone
	Very severe pneumonia: ceftriaxone (50mg/kg/day) + oxacillin (50mg/kg/day) and injectable dipyrone
Radiological examinations	1 chest radiography

*Drug treatment costs were estimated based on: (dose/kg x number of administrations/day x duration of treatment x price of one dose).

The length of stay at ward or ICU was the mean hospitalization days reported in selected records. As with medical record review based costing, daily hospital bed and medical fee costs were estimated by dividing respective SUS reimbursement rates by patient mean length of stay.

Drug prices listed by the national agencies BPS,^14^ CMED^15^ and *Associação Brasileira do Comércio Farmacêutico* (ABC Farma) were used.^16^ The remaining components were priced according to SIGTAP.^17^ Respiratory physical therapy was not included in therapeutic guidelines. As mentioned, oxygen supply, although recommended for hypoxemic children, was not listed in SUS tables and was therefore not considered.

### Costing based on reimbursement rates

Hospitalization rates practiced by SUS hospitals, or by private hospitals with contract with the SUS, are reimbursed via a package of professional and hospital services including meals, hospital room rates, hospital materials, drugs and ancillary diagnostic tests. This package has a fixed value defined according to diagnosis of each admission (based on ICD code indicated as diagnosis upon discharge of each patient). Reimbursement rates correspond to R$ 504,07/$Int. 554.47 (hospitalization due to pneumonia) and R$ 78,40/$Int. 86.24 (medical fees per hospitalization). A standard value reflecting minimum length of stay for a given diagnosis is paid.^19^ This amount is paid whenever a minimum of 50% of expected length of stay is actually completed (4 days for pneumonias cases; procedure 0303140151 – SIGTAP/DATASUS). Additional R$ 20,00 are paid for longer hospitalization time (from 9 days on).^17,19^

Copies of authorizations for hospital admission (AIH - *Autorizações de Internação Hospitalar*) were obtained from the Hospital Information System (SIH - *Sistema de Informação Hospitalar*) of SUS Information Technology Department (DATASUS - *Departamento de Informática do Sistema Único de Saúde*) via the Municipal Health Department of Goiânia. *Sistema Único de Saúde* reimbursement rates per patient, including hospital services, daily companion charge rates, professional services and respiratory physical therapy were identified and analyzed for hospitalization cost estimation on a per patient basis.

### Data analysis

A descriptive analysis of demographic and clinical profiles of pneumonia cases was performed. In each costing analysis, mean treatment costs and respective 95% confidence interval (95%CI) and standard deviation (SD) were calculated for severe and very severe pneumonia. Given the low numbers of very severe cases, only severe inpatient cases treated at wards were considered in comparative analysis of costs estimates. The Friedman test was used to investigate cost differences in the methods, via one-to-one comparisons, at a significance level of 0.05. Data were entered into Excel spreadsheets and statistical analyses were performed using the Statistical Package Social Sciences (SPSS) software version 23.

This study was approved by the Ethics Committee of *Hospital das Clínicas da Universidade Federal de Goiás*, Research Ethics Committee (CEP) protocol MHA/HC/UFG 174/09.

## RESULTS

### Study sample and health resource consumption

The clinical and demographic features of the 59 CAP inpatients are presented in table 1. The cases were classified according to severity, as follows: 52 (88%) patients with severe pneumonia, mean length of stay of 4 days (SD: 2.2; minimum, 2 days; maximum, 13 days) and 7 (12%) patients with very severe pneumonia, mean length of stay at ICU and hospital ward of 7 days (SD: 5.1; minimum, 3 days; maximum, 18 days). Hospital stay of patients with very severe pneumonia was significantly (p<0.0001) longer compared to patients with severe pneumonia that did not require admission to ICU.

**Table 1:** Clinicoepidemiological features of patients hospitalized with severe and very severe pneumonia

Variables	Severe pneumonia (n=52)	Very severe pneumonia (n=7)
Sex, n (%)		
Male	28 (47)	6 (10)
Female	24 (41)	1 (2)
Age (months), mean (SD)	14.8 (9.6)	9 (8.3)
Criterium to confirm diagnosis, n (%)		
Clinical	26 (44)	0
Radiological	26 (44)	7 (12)
Hospital stay (days), mean (SD)	4 (2.2)	7 (5.1)*
Outcome, n (%)		
Discharge	52 (88)	6 (10)
Death	0	1 (2)

* For patients with very severe pneumonia, the mean length of stay was 5 days at pediatric intensive care unit and 2 days in the ward, totaling up 7 days. SD: standard deviation.

Health resources and respective amounts utilized for treatment of 52 patients affected with severe pneumonia in this study are detailed in table 2. All patients received pediatric medical care during admission (mean of 5 medical visits/consultations; SD: 2.1). Only one patient was seen by an infectious disease specialist. Most commonly prescribed antibacterial drugs were ampicillin (n=42, mean administration time, 3.14 days; SD: 1.29) and ceftriaxone (n=15, mean administration time, 4.8 days; SD: 5.10). Antimicrobial drug combinations were given to 22 patients (ampicillin/amoxicillin, 17 patients; gentamicin/amoxicillin, 2 patients; ampicillin/amoxicillin/gentamicin, 2 patients; ceftriaxone/oxacillin, 1 patient). Ancillary drugs given were corticosteroids (23 patients), nasal decongestants (30 patients), bronchodilators (21 patients), gastric protectants (10 patients) and antipyretics (40 patients). Laboratory tests and imaging examinations were performed in 16 (31%) and 13 (25%) children, respectively. Three children underwent respiratory physical therapy (mean of 3.67 sessions; SD: 2.08) and 49 received nebulization (mean of 3.96 nebulizations/day; SD: 2.08).

**Table 2:** Type and amount of health resource used per hospitalized patients with severe pneumonia

Health resource	Prescription/amount/mean	Patients
	duration (SD)	n (%)
Medical visit (pediatrician)	5 visits (SD: 2.1)	52 (100)
Medical visit (infectious disease specialist)	1 visit	1 (2)
Antibacterial drugs		
Ampicillin (injectable)	Prescribed every 6 hours; 3.14 days (SD: 1.29)	42 (81)
Amoxicillin (oral)	Prescribed every 8 hours; 1.58 days (SD: 1.16)	15 (29)
Benzylpenicillin (injectable)	Prescribed every 6 hours; 2.2 days (SD: 1.30)	5 (10)
Cephalexine (oral)	Prescribed every 6 hours; 3 days	3 (6)
Cephalotine (injectable)	Prescribed every 6 hours; 4.50 days (SD: 1.87)	6 (11)
Ceftriaxone (injectable)	Prescribed every 12 hours; 4.8 days (SD: 5.10)	15 (29)
Gentamicin (injectable)	Prescribed every 12 hours; 4 days (SD: 3.54)	4 (15)
Oxacillin (injectable)	Prescribed every 6 hours; 6 days (SD: 1)	1 (2)
Other drug classes		
Dipirone (oral)	Prescribed every 6 hours; 1.5 days (SD: 0.58)	4 (8)
Dipyrone (injectable)	Prescribed every 6 hours; 1.65 days (SD: 0.95)	35 (67)
Paracetamol (oral)	Prescribed every 6 hours; 1 day	1 (2)
Bromopride (injectable)	Prescribed every 8 hours; 1 day	1 (2)
Bromopride (oral)	Prescribed every 8 hours; 1 day	1 (2)
Domperidone	Prescribed every 12 hours; 1.86 days (SD: 0.90)	4 (8)
Omeprazole (injectable)	Prescribed every 12 hours; 3.33 days (SD: 1.15)	2 (4)
Ranitidine (oral)	Prescribed every 12 hours; 9.5 days (SD: 2.12)	2 (4)
Dexametasone (injectable)	Prescribed every 8 hours; 2 days (SD: 1.41)	2 (4)
Hydroxyzine (injectable)	Prescribed every 6 hours; 2.97 days (SD: 1.85)	6 (11)
Prednisolone (oral)	Prescribed every 12 hours; 1.4 day (SD: 0.89)	11 (21)
Prednisolone (oral)	Prescribed every 24 hours; 2.14 days (SD: 1.21)	4 (8)
Acebrophylline (oral)	Prescribed every 12 hours; 2.09 days (SD: 1.45)	8 (15)
Acetylcysteine (oral)	Prescribed every 8 hours; 8 days (SD: 9.64)	9 (17)
Ambroxol hydrochloride (oral)	Prescribed every 12 hours; 3 days (SD: 1.26)	4 (8)
Naphazoline (nasal)	Prescribed every 4 hours; 3 days (SD: 2.41)	21 (40)
Saline solution (nasal)	Prescribed every 12 hours; 3 days (SD: 1.26)	9 (17)
Nebulization	3.96 nebulizations prescribed/day (SD: 2.08)	49 (94)
Saline solution/fenoterol	Prescribed every 5.8 hours (SD: 0.84)/3.49 days (SD: 1.88)	52 (100)
Saline solution/ beclometasone	Prescribed every 12 hours; 1.71 days (SD: 0.76)	19 (37)
Respiratory physical therapy	3.67 sessions prescribed (SD: 2.08)	3 (6)
Radiological examinations (X-ray)	0.35 examinations prescribed (SD: 0.62)	13 (25)
Laboratory tests		
Complete blood count	Prescribed mean, 0.38 (SD: 0.75)	13 (25)
C-reactive protein	Prescribed mean, 0.60 (SD: 1.06)	9 (17)
Serum sodium	Prescribed mean, 0.26 (SD: 0.47)	4 (8)
Serum potassium	Prescribed mean, 0.40 (SD: 0.51)	6 (11)
Serum calcium	Prescribed mean, 0.06 (SD: 0.24)	3 (6)
Serum magnesium	Prescribed mean, 0.06 (SD: 0.24)	3 (6)
Chlorides	Prescribed mean, 0.33 (SD: 0.49)	5 (10)
Arterial blood gas analysis	Prescribed mean, 0.07 (SD: 0.26)	1 (2)

SD: standard deviation.

### Costs of pneumonia estimated by different methods

Very severe pneumonia treatment costs were significantly (p<0.001) higher compared to severe pneumonia, regardless of the costing method employed. Severe pneumonia treatment costs varied across costing methods, with mean costs corresponding to R$ 780,70 (medical record review; 95%CI: 674-885), R$ 641,90 (therapeutic guideline; 95%CI: 639-642) and R$ 594,80 (SUS reimbursement; 95%CI: 566-627) (Table 3).

**Table 3:** Inpatient severe and very severe pneumonia treatment *Sistema Único de Saúde* costs estimated via different costing methods

	Estimated cost (R$)	95% confidence interval	Standard deviation
Severe pneumonia (n=52)			
Costing based on medical record	780,70	674-885	393.50
Costing based on therapeutic guidelines	641,90	639-642	2.38
Costing based on reimbursement rates	594,80	566-627	108.30
Very severe pneumonia (n=7)			
Costing based on medical record	3.569,20	1,269-5,807	2,453
Costing based on therapeutic guidelines	3.369,40	3,263-3,476	8
Costing based on reimbursement rates	3.175,00	682-5,667	2,719

Based on medical record review, daily cost estimates for the mean length of stay in this study (4 days) amounted to R$ 126,00/$Int. 138.60 and R$ 19,58/$Int. 21.53 (hospital ward services and medical fees respectively). Mean overall costs corresponded to R$ 780,70 (SD: 393.5; minimum, 343.14; maximum, 2,415.96)/$Int. 858.77, with R$ 750,10/$Int. 825) and R$ 30,60/$Int. 33.6 reflecting medical and non-medical services respectively. Ward hospitalization costs amounted to R$ 507,20 (SD: 269; minimum, 250; maximum, 1,630)/$Int. 557.90, while drugs costs amounted to R$ 159,00 (SD: 68, minimum 63.50, maximum 387.30)/ $Int. 174.90. Antibacterial and ancillary drug costs accounted for 40% and 60% (R$ 64/$Int. 70.4 and R$ 95/$Int. 104.5) of total drug costs respectively.

Therapeutic guideline based costing yielded mean overall cost estimates of R$ 641,90 (SD: 2.38; minimum, 635.20; maximum, 645)/$Int. 706, with R$ 609,90/$Int. 670.90 and R$ 32,00/$Int. 35.20 reflecting medical and non-medical costs respectively. Mean values estimated via reimbursement based costing corresponded to R$ 594,80 (SD: 108.30; minimum, 201.07; maximum 847.64)/$Int. 654.28, with R$ 565,50/$Int. 622 and R$ 29,30/$Int. 32.23 reflecting medical and non-medical costs respectively (Table 4).

**Table 4:** Treatment cost of severe pneumonia *Sistema Único de Saúde* per cost component across different costing methods

Severe pneumonia	Costing	Costing	Costing
	(medical record)	(therapeutic guidelines)	(reimbursement)
	R$ (%)	R$ (%)	R$ (%)
Hospital services	507,20 (65.0)	504,00 (78.5)	487,60 (82.0)
Professional services	75,30 (9.6)	78,40 (12.2)	76,80 (12.9)
Drugs	159,00 (20.4)	18,00 (2.8)	------*
Radiological examinations	4,30 (0.6)	9,50 (1.5)	-------*
Laboratory tests	3,30 (0.4)	------†	-------*
Physical therapy	1,00 (0.1)	-----†	1,10 (0.2)
Companion charge rates	30,60 (3.9)	32,00 (5.0)	29,30 (4.9)
Final mean cost	780,70 (858.65)	641,90 (705.00)	594,80 (654.40)
R$ ($Int)			

*Included in hospital service reimbursement rates; ^†^not listed in guidelines.

Estimated very severe pneumonia treatment costs varied across costing methods. Cost estimates obtained via micro-costing methods (medical record review or therapeutic guidelines) did not differ significantly (p=0.405), while the estimated cost for repayment was significantly lower than the costs estimated by therapeutic guideline (p<0.001) or medical record review (p=0.006) basead cost estimates.

## DISCUSSION

There is evidence to suggest that length of stay has a significant impact on overall hospital costs,^20^ including pneumonia or pneumococcal disease treatment.^21,22^Mean length of stay in this study (4 days) is within expected SUS time frame for pneumonia treatment^17^ and is consistent with WHO (World Health Organization) recommendations of 5 days, and with data from a recent global systematic review addressing treatment costs of pneumonia in children (mean of 5.8 days).^23^

The hospital costs of severe pneumonia in children estimated in this study reflect data from Latin American studies (R$ 721,36 to R$ 1.488,26, top-down gross-costing^24^and up to R$ 1.373,53^25^ or R$ 2.957,34,^26^ combined bottom-up/top-down costing) following adjustment for inflation in the country of origin and conversion to American dollars (PPP 2010)^6^ to *reals* (2011). As expected, costs estimates based on reimbursement rates were significantly lower compared to costs estimates based on medical record review.

Cost-of-illness estimates are vital for economic burden, health status and health intervention cost-effectiveness studies. Brazilian studies comparing cost estimates based on SUS reimbursement rates and other inpatient treatment costing methods are scarce. However, evidence suggests that reimbursement rate based costing underestimates true hospitalization costs.^27^ Cost estimates based on reimbursement rates differed significantly from estimates based on therapeutic guidelines or medical record review in this study. This finding supports evidence suggesting that costing methods may significantly impact costing studies results.^7,10^

Costs estimate differences may reflect different costing methods, method-specific accuracy regarding cost component identification in gross-costing or micro-costing, and cost component valuation (top-down or bottom-up approach).^7^ Methodological differences may therefore explain cost estimate heterogeneity across Latin American costing studies.

Studies comparing costing methods are scarce. A study by Swindle et al.,^28^ investigating the value of combined gross-costing and micro-costing to assess variations in utilization of essential hospital resources failed to reveal differences between top-down and bottom-up approaches. Wordsworth et al.,^29^ compared top-down and bottom-up cost estimates but did not explore the gross-costing method. Tan et al.,^20^ compared the reliability of overall hospital service cost estimates based on bottom-up/micro-costing, top-down/microcosting or gross-costing analysis in the Netherlands. This study is the first to assess inpatient costs of a single patient sample via three different costing methods.

Bottom-up micro-costing (*i.e*., costing estimates based on medical record review) is thought to be the most accurate costing method and the gold standard.^7^ However, this methodology is difficult to apply, labor intensive, time consuming and expensive. The top-down approach combined with micro-costing or gross-costing may represent an easier and less expensive alternative.^7,9^ Still, evidence suggests that bottom-up micro-costing is the method of choice to estimate the cost of components with greater impact on overall daily hospital stay costs.^20,29^

The hospitalization costs of severe pneumonia patients paid by SUS and estimated via top-down (therapeutic guidelines) or bottom-up (medical record review) micro-costing did not differ significantly in this study, suggesting that top-down micro-costing may be a more practical and less expensive costing alternative.^20^ Also, therapeutic guideline based costing is easy to apply within the SUS financing structure and allows cost estimation of a wider range of components compared to reimbursement rate based costing.

This study has several limitations. First, the low number (n=7) of very severe pneumonia cases in the sample limited comparing the three methods in this group of patients. Further costing studies with an adequate number of pneumonia cases requiring ICU hospitalization are therefore warranted. Also, the average daily hospital stay cost of R$ 126,00 adopted in this study may have translated into increased therapeutic guideline and medical record based cost estimates.

## CONCLUSION

SUS cost estimates obtained via top-down (based on therapeutic guidelines) or bottom-up (based on medical record review) micro-costing did not differ significantly in this study, suggesting that top-down micro-costing may be an alternative to micro-costing based on medical record review for severe pneumonia treatment cost estimation. Studies comparing different costing methods and focusing on different diseases are warranted to support our findings. These results may subsidize economic evaluation of interventions for prevention and control.
